# Melatonin regulates rabbit sperm motility and kinematics via the MT1/PKC signaling pathway

**DOI:** 10.5713/ab.24.0593

**Published:** 2025-02-27

**Authors:** Chongchong Wang, Yanyan Zhang, Shiwen He, Biao Jiang, Jinglei Huang, Hui Peng

**Affiliations:** 1 School of Tropical Agriculture and Forestry, Hainan University, Hainan, China; 2 College of Animal Science, Fujian Agriculture and Forestry University, Fujian, China

**Keywords:** Melatonin, Melatonin Receptors, PKC Signaling Pathway, Rabbit, Sperm Kinematics

## Abstract

**Objective:**

Melatonin, a highly conserved molecule, plays an essential role in various physiological functions. Research suggests that incorporating melatonin into semen extender enhances livestock sperm viability. However, the effect of melatonin on rabbit sperm and the molecular mechanisms underlying melatonin-regulated rabbit sperm motility and kinematics remain unclear. This study aimed to reveal the molecular mechanism by which melatonin regulates rabbit sperm motility and kinematics.

**Methods:**

This study investigated the expression and localization of melatonin-related proteins in rabbit testis and epididymis. Rabbit sperm was incubated at different concentrations of melatonin for 60 min or 90 min at 37°C, followed by an evaluation of sperm motility parameters using IVOS II computer assisted sperm analyzer system (CASA). Then we examined the integrity of the sperm plasma membrane, mitochondrial membrane potentials and intracellular reactive oxygen species. Furthermore, melatonin receptor antagonists were added to the extender and investigated the involvement of the melatonin receptors in the regulation of sperm motility parameters. We carried out phosphoproteomics analysis and verified regulation of rabbit sperm kinematics by melatonin via the inhibition of potential signaling pathway.

**Results:**

Melatonin-related proteins were expressed and localized in testes and epididymis of rabbits. Melatonin (5 mM) markedly increased rabbit sperm motility, kinematics, and overall sperm quality. Melatonin regulated rabbit sperm motility and kinematics via the MT1 receptor. Phosphoproteomics combined with GPS 5.0 software revealed the potential signaling pathway by which melatonin regulates sperm kinematics. Moreover, the inhibition of protein kinase C (PKC) markedly reduced rabbit sperm kinematics, whereas the inhibition of ERK1/2, p38 MAPK, PKG and JNKs using kinase inhibitors did not result in obvious changes in sperm kinematics. In addition, inhibition of the MT1 receptor significantly weakened PKC activity, suggesting that PKC is downstream of MT1.

**Conclusion:**

We conclude that melatonin regulates rabbit sperm kinematics through the MT1/PKC signaling pathway.

## INTRODUCTION

In mammals, melatonin is mainly synthesized and secreted in the pineal gland and retina according to a circadian rhythm. Apart from this secretion, it can also be produced in other body fluids and tissues with different distributions. The extensive distribution of melatonin in different tissues suggests that this molecule plays key roles in diverse biological processes, including oxidative stress defense [1], scavenging of reactive oxygen species (ROS) and reactive nitrogen species (RNS) [2], immune regulation [3] and mammalian reproduction [4].

Exogenous melatonin can regulate ovarian activities, delay fertility decline [5], improve oocyte maturation [6] and early embryonic development [7]. Melatonin implants in rams could increase testicular function [8] and improve testicular hemodynamics and sperm quality [9]. Meanwhile, melatonin was present in ram seminal plasma and human semen and correlated with male infertility [10,11].

Recently, studies have reported that in vitro treatment with melatonin could enhance hyperactivation of hamster sperm [12], improve fresh semen motility variables [13], improve human sperm motility [14], reduce lipid peroxidation in stallion spermatozoa [15], and affect boar sperm motility and adhesiveness [16]. Meanwhile, supplementation with melatonin could raise the proportion of live sperm in boar semen under the condition of storage at 17°C [17] and strengthen ram sperm quality during storage at 4°C [18]. Furthermore, the addition of melatonin benefits the cryopreservation of semen in rabbits [19]. The positive effects of melatonin on sperm are partially associated with its free radical scavenging properties and its ability to decrease oxidative stress levels. In addition, melatonin also carry out its physiological functions via the membrane receptors MT1 in human and boar spermatozoa [20,21] and MT2 in ram sperm [22]. Although the beneficial effects of melatonin in sperm in humans and livestock have been reported, the effects of melatonin on rabbit sperm as well as the underlying mechanism were uncovered.

Melatonin was synthesized from tryptophan by several enzymes including aralkylamine N-acetyltransferase (AANAT) and acetylserotonin O-methyltransferase (ASMT), which are rate-limiting enzyme and key enzyme to convert N-acetylserotonin to melatonin, respectively. The physiological function of melatonin often mediated by two G-protein-coupled melatonin receptor 1A (MT1) and melatonin receptor 1B (MT2). Besides, melatonin receptor 1C (MT3) receptor was found in galliform birds, while this receptor was considered as quinone reductase, also known as N-ribosyldihydronicotinamide:quinone dehydrogenase 2 (NQO2), in mammals. Therefore, in this study, the expression and localization of melatonin-related proteins (AANAT, ASMT, MT1, MT2 and NQO2), the effects of melatonin on rabbit sperm motility and kinematics and quality and the potential mechanism by which melatonin regulates rabbit sperm kinematics were investigated.

## MATERIALS AND METHODS

### Animal care

All animal procedures were ethically approved by the Animal Care and Use Commission of the School of Tropical Agriculture and Forestry, Hainan University (HNUAUCC-2022-00090).

### Animals and diets

A total of twenty 1-year-old Fujian yellow rabbit bucks were utilized for this study. Each rabbit was individually housed in a cage and kept under natural daylight conditions. They received a standard commercial diet and had ad libitum access to water at the Laboratory Animal Facility of the School of Tropical Agriculture and Forestry, Hainan University.

### Chemicals

All chemicals and reagents were sourced from Sigma-Aldrich (St. Louis, MO, USA) unless otherwise specified.

### Western blot

Rabbit tissues (ovary, uterus, epididymis, and testis; n = 3) from 1-year-old Fujian yellow rabbits were lysed using RIPA lysis buffer (Beyotime Biotechnology, Shanghai, China) supplemented with a proteinase inhibitor cocktail (Roche, Mannheim, Germany). Subsequently, 20 μg of proteins per lane were separated by sodium dodecyl sulfate polyacrylamide gel electrophoresis, transferred to PVDF membranes (Millipore, Bedford, MA, USA), and blocked with 5% dry skim milk. The membranes were then incubated overnight at 4°C with primary antibodies, which were thoroughly assayed for specificity, against AANAT (1:500, anti-AANAT antibody; Sigma-Aldrich), ASMT (1:500, anti-ASMT antibody; Sigma-Aldrich), MT1 (1:500, MEL-1A-R Antibody; Santa Cruz Biotechnology, Dallas, TX, USA), MT2 (1:500, Anti-MTR1B antibody; Sigma-Aldrich), and NQO2 (1:200, Anti-NQO2 antibody; Sigma-Aldrich). After washing, the blots were probed with a horseradish peroxidase-conjugated secondary antibody (1:2,000; Pierce, Rockford, IL, USA) and visualized using an enhanced chemiluminescence Advanced Western Blotting Detection System (Pierce). GAPDH served as a loading control (1:1,000; Santa Cruz Biotechnology).

### Immunofluorescence

Epididymis and testes obtained from 1-year-old Fujian yellow rabbit (n = 3) were fixed with 4% paraformaldehyde, paraffin-embedded, and sectioned at 6 μm thicknesses. Deparaffinization, rehydration, and antigen retrieval were performed before blocking and overnight incubation with primary antibodies against AANAT (1:100, anti-AANAT antibody; Sigma-Aldrich), ASMT (1:100, anti-ASMT antibody; Sigma-Aldrich), MT1 (1:50, MEL-1A-R antibody; Santa Cruz Biotechnology), MT2 (1:100, anti-MTR1B antibody; Sigma-Aldrich), and NQO2 (1:100, anti-NQO2 antibody; Sigma-Aldrich). After washing, sections were incubated with an Alexa Fluor 488-conjugated goat anti-rabbit secondary antibody (Beyotime Biotechnology) and counterstained with 4′,6-diamidino-2-phenylindole (DAPI; Beyotime Biotechnology). Imaging was carried out using an Eclipse Ti-S microscope equipped with a 198 DS-Ri1 digital camera (Nikon, Tokyo, Japan). Negative controls (NC) were established by omitting primary antibodies.

### Semen collection and evaluation

Semen was collected at least from five rabbits every time using an artificial vagina. The semen was immediately pooled (n = 6) and diluted at a ratio of 1:4 with a Tris-citrate-glucose extender (TCG) at pH 6.8 containing 250 mM Tris, 87.5 mM citric acid, 69 mM glucose, 100 million IU penicillin sodium and 100 million IU streptomycin sulfate. The diluted semen was transferred to the laboratory within 30 min for further evaluation. In the lab, the diluted semen was re-extended with TCG at 1:2 ratio. Then 2 microliters of the final diluted semen were added to a prewarmed eight-chamber Leja slide and assessed by a CASA system instead of microscope evaluation. Only semen samples with over 80% motile sperm were used in this study.

### Kinetic parameters assessment

Aliquots of 1 mL rabbit sperm were incubated with 0.1 mM, 1 mM, 5 mM and 10 mM melatonin for 60 min and 90 min respectively, then 2 μL semen were added to a prewarmed eight-chamber Leja slide and assessed by IVOS II CASA. Kinetic parameters, including the percentage of total motile (TM) and progressive motile (PM) sperm, average path velocity (VAP), curvilinear velocity (VCL), straight-line velocity (VSL), straightness index (STR), amplitude of lateral head displacement (ALH), and wobble (WOB), were analyzed. The group containing identical DMSO concentration was used as the NC. The group with DMSO-free and melatonin-free was used as the blank control.

### Sperm plasma membrane integrity evaluation

Rabbit sperm (n = 6) were incubated with either 0 mM or 5 mM melatonin at 37°C for 60 min. Afterwards, sperm plasma membrane integrity was assessed using Viadent stain (Hamilton-Thorne Biosciences, Beverly, MA, USA). Briefly, Viadent stain was diluted in extender to 10 μg/mL concentration in a Viadent tube, gently vortexed for 5 seconds and maintained at 37°C. 500 μL aliquots of rabbit sperm at 3 × 10^7^ sperm/mL were mixed with 500 μL of diluted Viadent stain. The stained sperm sample was then incubated at 37°C for 2 min. Following that, 2 μL of the stained sample was loaded into a prewarmed Leja slide and analyzed under blue light with a VIADENT filter by a CASA system.

### Mitochondrial membrane potential assay

To assess mitochondrial membrane potential changes (ΔΨm), we used a mitochondrial membrane potential assay kit with JC-1 (Beyotime Biotechnology, Shanghai, China) according to the manufacturer’s protocol. In brief, 500 μL aliquots of rabbit sperm from control (n = 6) and melatonin-treated groups (n = 6) were centrifuged and resuspended in extender. The sperm samples were mixed with JC-1 working solution and incubated. After washing with JC-1 staining buffer, the stained sperm were examined under a fluorescence microscope (Nikon).

### Intracellular reactive oxygen species evaluation

To evaluate intracellular ROS, a ROS assay kit (Beyotime Biotechnology, Shanghai, China) was utilized based on the manufacturer’s guidelines. Control and melatonin-treated sperm samples (n = 6) were incubated with DCFH-DA solution to label intracellular ROS. After washing off excess dye, ROS levels were indicated by fluorescence intensity and quantified by image analysis software (National Institutes of Health, Bethesda, Maryland, USA).

### Melatonin receptor inhibition

To study melatonin receptor inhibition, competitive antagonists luzindole (Santa Cruz Biotechnology) and 4P-PDOT (Santa Cruz Biotechnology) were used. 0.1 mM Luzindole and 0.1 μM 4P-PDOT have proved effective concentrations [23,24]. The experimental groups in the antagonist assays were as follows: (1) DMSO supplementation as a NC (n = 6); (2) a group exposed to 5 mM melatonin (MLT; n = 6); (3) a group incubated with 0.1 mM luzindole (n = 6); (4) a group incubated with 0.1 μM 4P-PDOT (n = 6); (5) a group treated with 5 mM melatonin plus 0.1 mM luzindole (n = 6); and (6) a group treated with 5 mM melatonin plus 0.1 μM 4P-PDOT (n = 6). In each treatment group, rabbit sperm was incubated for 60 min at 37°C, and then motility was assessed by IVOS II CASA.

### Phosphoproteomics of rabbit sperm

For phosphoproteomics analysis of rabbit sperm, the samples were sonicated three times on ice using a high-intensity ultrasonic processor (Scientz) in lysis buffer. The lysates were then subjected to centrifugation at 12,000×g at 4°C for 10 min. The protein concentration was determined using a BCA kit, and the protein solution was digested with trypsin. After digestion, the peptide was desalted using a Strata X C18 SPE column (Phenomenex, Torrance, CA, USA) and vacuum-dried. For rabbit sperm phosphoproteomics, peptides were enriched with phosphopeptides (with serine, threonine, or tyrosine phosphorylation sites) through a combination of immobilized metal affinity chromatography (IMAC) beads and TiO2 beads. The supernatant containing phosphopeptides was collected and lyophilized for LC-MS/MS analysis.

The resulting MS/MS data were processed using MaxQuant software (version 1.5.2.8). False discovery rates (FDRs) were adjusted to less than 1%, and the minimum score for modified peptides was set to more than 40. Screening criteria for significantly differentially expressed (DE) proteins or sites were based on a combination of FDR less than 0.05 and an absolute fold change larger than 1.2. The fold changes of phosphorylated proteins were normalized to the protein fold change in the proteome.

Proteins underwent classification based on Gene Ontology (GO) annotation. A two-tailed Fisher’s exact test was applied to assess the enrichment of differentially modified proteins against all identified proteins for each category. GO terms with a corrected p value less than 0.05 were regarded as significant. The Encyclopedia of Genes and Genomes (KEGG) database was utilized to identify enriched pathways by applying a two-tailed Fisher’s exact test to test the enrichment of differentially modified proteins against all identified proteins. Pathways with a corrected p value less than 0.05 were considered significant and were further categorized hierarchically according to the KEGG website.

### Prediction of kinase-substrate regulations

The GPS 5.0 software (http://gps.biocuckoo.cn/) was utilized for the prediction of kinase-substrate regulations based on short linear motifs (SLMs) around phosphorylation sites (p-sites). The kinase proteins were compared with the IEKPD2.0 database (http://iekpd.biocuckoo.org/index.php).

### Inhibition of the potential signaling pathway

Inhibitors of protein kinase C (PKC; Ro31-8220), ERK1/2 (FR180204), p38 MAPK (SB203580), PKG (KT5823), and JNKs (SP600125) were used to verify the signaling pathways through which melatonin regulates sperm kinematics. The inhibitors were added to rabbit sperm, incubated, and sperm kinematics were detected by IVOS II CASA.

### PKC activity assay

To assess PKC activity, luzindole or 4P-PDOT were incubated with 5 mM melatonin and rabbit sperm at 37°C for 60 min. After incubation, the sperm samples (n = 6) were collected and PKC activity was measured using a PKC Kinase Activity Assay Kit (ab139437; Abcam, Cambridge, UK) following the manufacturer’s protocol. Briefly, the kinase reaction was performed in the kit, then absorbance at 450 nm was recorded using a microplate reader as an indicator of PKC enzymatic activity in the sperm samples under different treatment conditions.

### Statistical analysis

All data were analyzed using one-way ANOVA. Tukey’s multiple comparisons test was performed using SPSS version 22 (IBM-SPSS Inc., Chicago, IL, USA). Results are presented as mean±standard error of the mean. Statistical significance was considered at p<0.05.

## RESULTS

### Expression and localization of melatonin-related proteins in rabbit tissues

**Figure 1:**
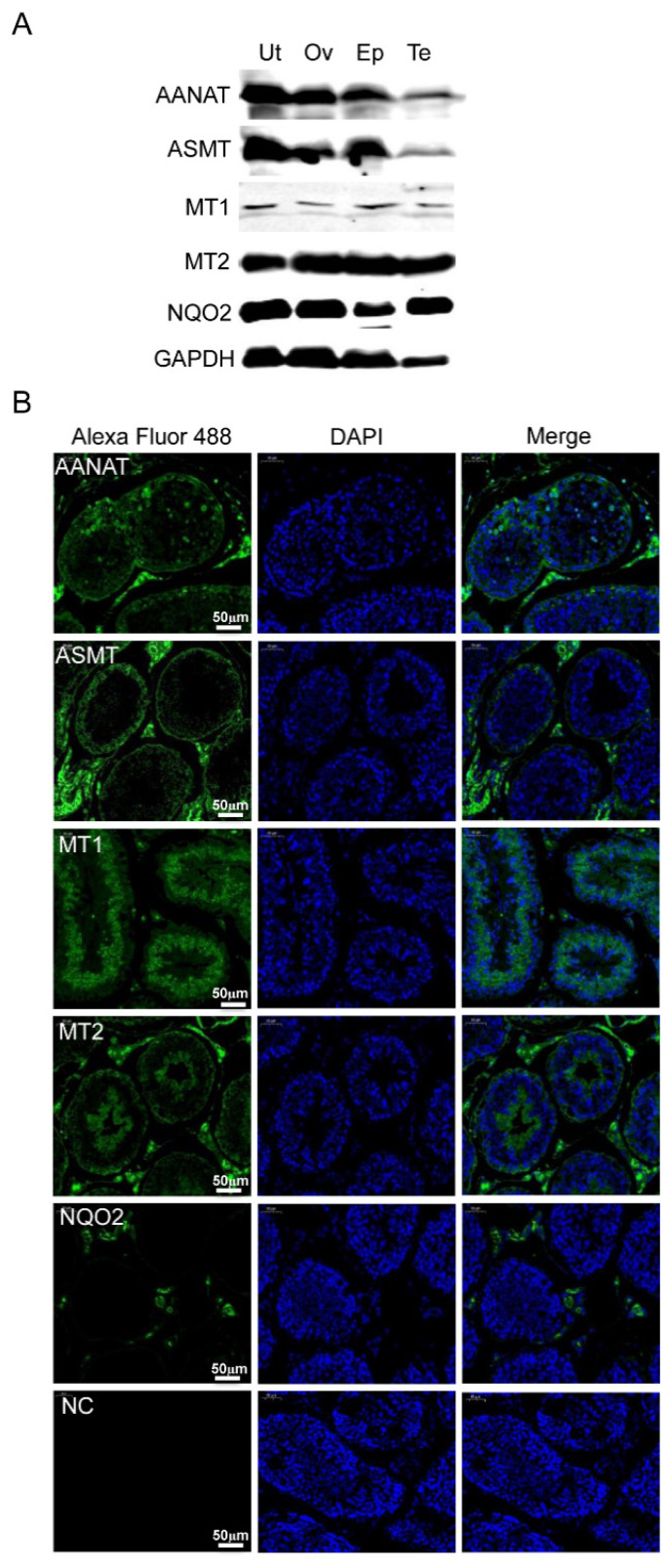
Expression and localization of melatonin-related proteins in rabbit tissues. (A) Western blot of protein lysates isolated from rabbit uterus (Ut), ovary (Ov), epididymis (Ep) and testis (Te). GAPDH was used as a loading control. (B) Rabbit testis was fixed, permeabilized, stained with AANAT-, ASMT-, MT1-, MT2- and NQO2-specific antibodies (green), and counterstained with DAPI to show DNA (blue). The white bar indicates 50 μm. DAPI, 4′,6-diamidino-2-phenylindole; NC, negative control.

**Figure 2:**
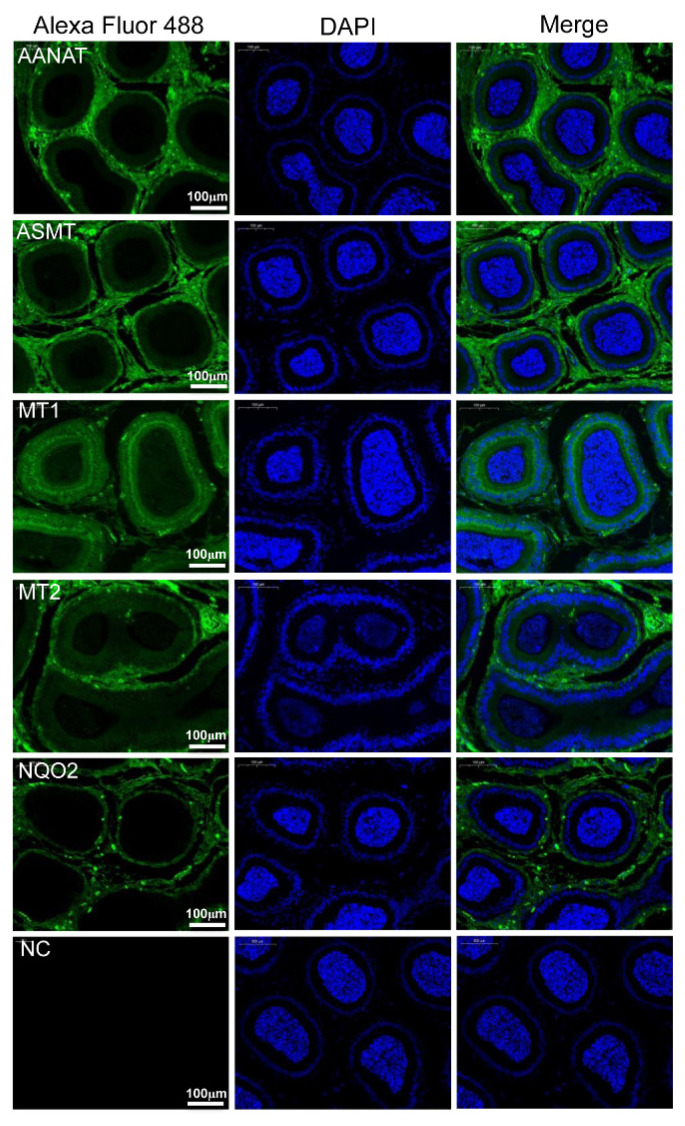
Localization of melatonin-related proteins in rabbit epididymis. Rabbit epididymis was fixed, permeabilized, stained with AANAT-, ASMT-, MT1-, MT2- and NQO2-specific antibodies (green), and counterstained with DAPI to show DNA (blue). The white bar indicates 100 μm. DAPI, 4′,6-diamidino-2-phenylindole; NC, negative control.

To elucidate the expression of melatonin-related proteins in rabbit tissues, western blotting was performed. The results indicated that AANAT, ASMT, MT1, MT2 and NQO2 proteins are present in the rabbit uterus, ovary, epididymis and testes (Figure 1A). In the testes, indirect immunofluorescence assays revealed that AANAT, ASMT, MT1 and MT2 proteins were mainly localized in Leydig cells and reproductive epithelium, including Sertoli cells and germ cells, while NQO2 protein was primarily localized in Leydig cells (Figure 1B). In the epididymis, these proteins were chiefly distributed in epididymal interstitial cells and epithelial cells (Figure 2).

### Melatonin enhanced rabbit sperm motility and kinematics

To investigate the potential positive influence of melatonin on rabbit sperm motility parameters, we exposed the sperm to varying concentrations of melatonin for durations of 60 min and 90 min at 37°C, subsequently evaluating sperm motility and kinematics. As summarized in Tables 1, 2, incubation with 5 mM melatonin significantly improved rabbit sperm kinetic parameters such as TM, PM, VAP, VCL, VSL and ALH (p<0.05) compared with treatment with 0.1 mM, 1 mM, and 10 mM melatonin groups and the control groups. Meanwhile, treatment with 10 mM melatonin markedly decreased sperm kinetic parameters such as TM, PM, VAP, VCL, VSL and ALH compared with treatment with 5 mM melatonin (p<0.05). In addition, under treatment with 5 mM melatonin, rabbit sperm kinetic parameters such as TM, PM, VAP, VCL, VSL, STR and WOB after incubation for 60 min were superior to those after incubation for 90 min.

### Melatonin improved rabbit sperm quality

**Figure 3:**
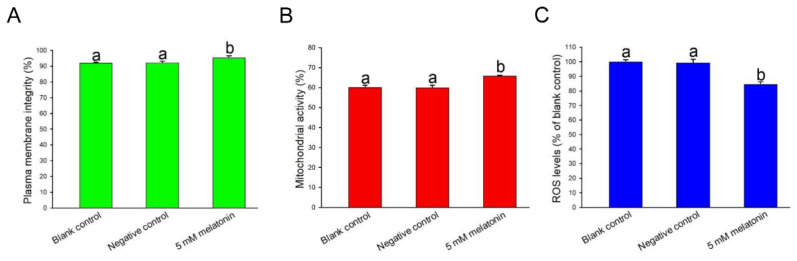
Melatonin supplementation affects plasma membrane integrity (A), mitochondrial activity (B) and ROS levels (C) in rabbit sperm. ^a,b^ Columns with different lowercase letters differ significantly (p<0.05). ROS, reactive oxygen species.

After 60 min of incubation, 5 mM melatonin sharply increased sperm plasma membrane integrity when stained with Viadent (p<0.05) compared with the blank and NCs (Figure 3A). Furthermore, the proportion of sperm exhibiting elevated mitochondrial membrane potentials significantly increased following treatment with 5 mM melatonin (p<0.05) (Figure 3B). Moreover, 5 mM melatonin significantly decreased the level of intracellular ROS compared with the controls (p<0.05) (Figure 3C).

### Melatonin increased rabbit sperm motility and kinematics via the MT1 receptor

**Figure 4:**
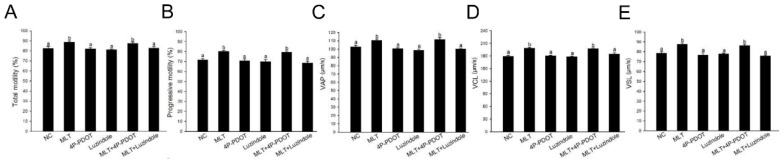
Effects of inhibition of melatonin receptors on the rabbit sperm TM (A), PM (B), VAP (C), VCL (D) and VSL (E). ^a,b^ Columns with different lowercase letters differ significantly (p<0.05). TM, the percentage of total motile sperm; PM, progressive motile sperm; VAP, average path velocity; VCL, curvilinear velocity; VSL, straight-line velocity.

As mentioned above, 5 mM melatonin increased rabbit sperm motility and kinematics. To delineate the favorable impact, we explored potential correlations between melatonin receptor inhibition and sperm motility parameters, encompassing TM, PM, VAP, VCL and VSL. Treatment with the MT2 specific antagonist 4P-PDOT (0.1 μM) plus melatonin (5 mM) did not impact rabbit sperm motility and kinematics compared with the 5 mM melatonin group, while incubation with the MT1 and MT2 competitive antagonist luzindole (0.1 mM) plus melatonin (5 mM) significantly decreased rabbit sperm motility and kinematics compared with the 5 mM melatonin group (Figures 4A–4E). These findings validate that the advantageous effects of melatonin on rabbit sperm motility and kinematics are mediated through the MT1 receptor.

### Phosphoproteomics analysis of rabbit sperm treated with melatonin

**Figure 5:**
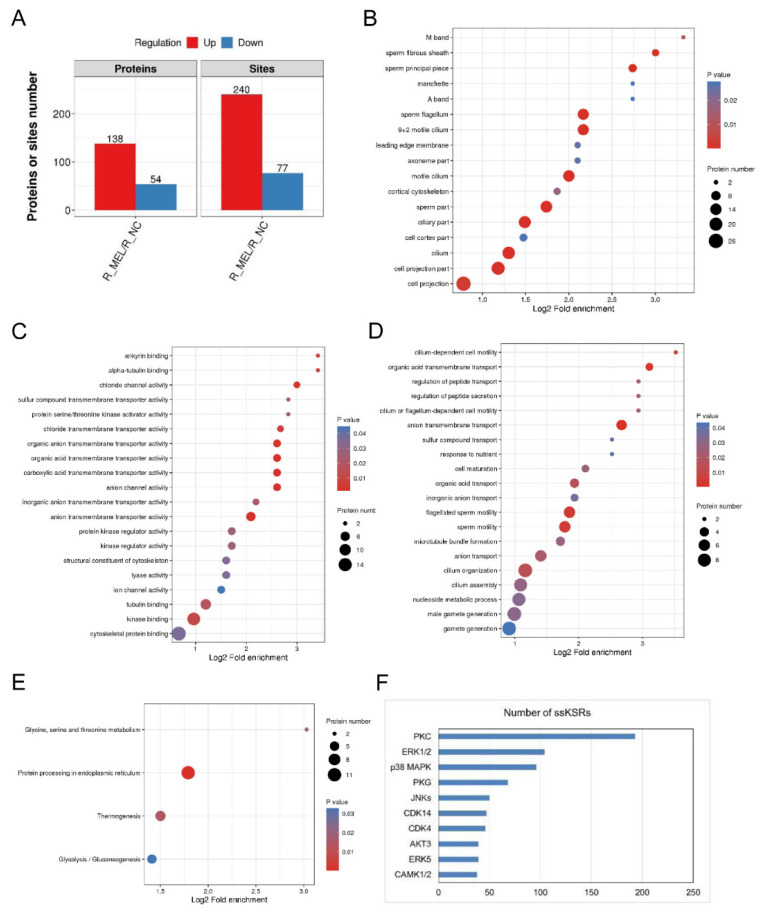
Phosphoproteomics analysis of rabbit sperm incubated with 0 mM or 5 mM melatonin for 1 h at 37°C. (A) Numbers of DE phosphorylated proteins and sites in R_MEL versus R_NC. (B–D) Gene Ontology (GO) enrichment analysis of DE phosphorylated proteins, which shows the response of the cellular component, molecular function and biological processes to melatonin treatment. (E) KEGG pathways of DE phosphorylated proteins, and the black dots represent the number of DE phosphorylated proteins. (F) Top 10 kinase families/subfamilies with the most ssKSRs predicted by GPS 5.0 software based on phosphoproteomic data. DE, differentially expressed; KEGG, Kyoto encyclopedia of genes and genomes.

To further study the molecular mechanism of the effects of melatonin on rabbit sperm kinematics, we incubated rabbit sperm with DMSO (R_NC) and 5 mM melatonin (R_MEL) and then collected them to carry out phosphoproteomics analysis. A total of 6201 phosphorylated sites were identified in the phosphoproteome, corresponding to 2092 phosphoproteins. The data were further filtered through a criterion of localization probability (larger than 0.75), and 4900 phosphorylated sites (corresponding to 1931 phosphoproteins) were identified. Using a combined criteria of FDR (less than 0.05) and absolute fold change (larger than 1.2), 240 upregulated sites (R_MEL/R_NC; corresponding to 138 phosphoproteins) and 77 downregulated sites (corresponding to 54 phosphoproteins) were found to be DE significantly (Figure 5A).

GO analysis indicated that the cellular components of the DE phosphorylated proteins were sperm flagellum, 9+2 motile cilium, motile cilium, sperm part and sperm principal piece (Figure 5B). The molecular function of the DE phosphorylated proteins was anion/organic acid transmembrane transporter activity, anion/chloride channel activity, protein kinase regulator activity and kinase binding (Figure 5C). The phosphoproteins for biological process were anion/organic acid transmembrane transport, flagellated sperm motility, sperm motility, cilium-dependent cell motility and cilium organization (Figure 5D). Furthermore, the KEGG database showed that most melatonin-altered proteins were mainly enriched in protein processing in the endoplasmic reticulum (ocu04141), thermogenesis (ocu04714) and glycolysis/gluconeogenesis (ocu00010) (Figure 5E).

### Melatonin regulates rabbit sperm kinematics through the MT1/PKC signaling pathway

To unveil the determined signaling pathway regulating sperm kinematics mediated by melatonin, phosphoproteomics data combined with GPS 5.0 software were employed to effectively screen upstream regulatory kinases and predict potential protein kinases [25,26]. Using this method, we identified 1652 site-specific kinase–substrate relations (ssKSRs) based on 132 protein kinases predicted by GPS 5.0 software. Among the 132 identified protein kinases, the top five kinase families/subfamilies with the most ssKSRs were PKC, ERK1/2, p38 MAPK, PKG and JNKs (Figure 5F). This result suggests that melatonin regulates sperm kinematics probably by affecting the activity of these kinases and their mediated signaling pathway.

**Figure 6:**
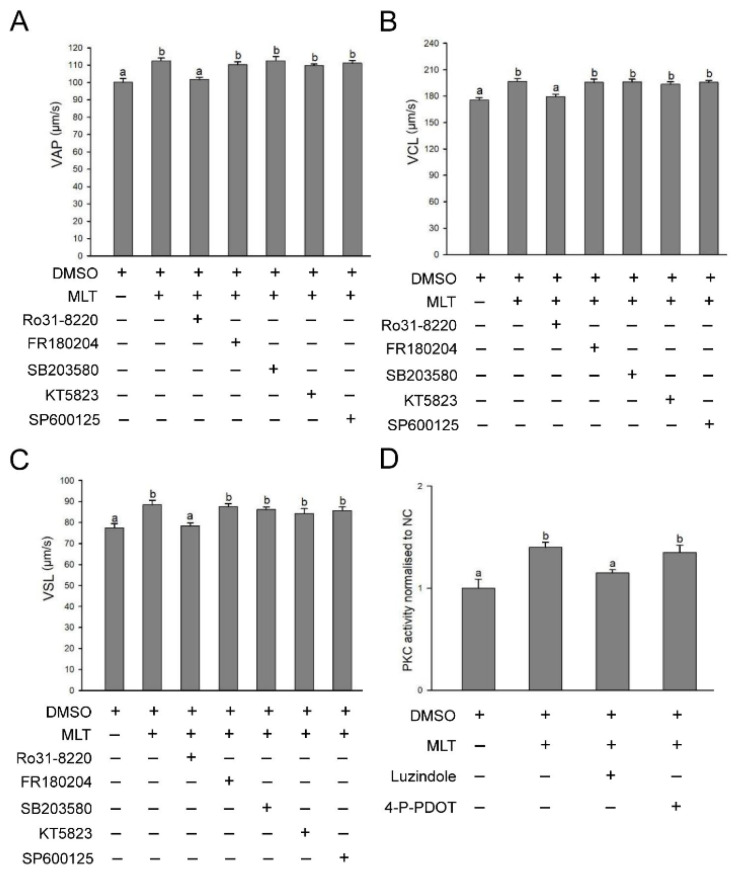
Antagonists of potential kinases verify the signaling pathway by which melatonin regulates sperm kinematics. (A–C) Effects of inhibition of potential kinases on rabbit sperm VAP, VCL and VSL. (D) A PKC activity assay was performed on rabbit sperm treated with luzindole or 4P-PDOT and 5 mM melatonin. ^a.b^ Columns with different lowercase letters differ significantly (p<0.05). VAP, average path velocity; VCL, curvilinear velocity; VSL, straight-line velocity.

To confirm the above hypothesis, PKC, ERK1/2, p38 MAPK, PKG and JNK inhibitors were added to rabbit sperm, and sperm kinematics were detected. In terms of VAP, VCL and VSL, inhibition of PKC with Ro31-8220 sharply reduced rabbit sperm kinematics, while inhibition of ERK1/2, p38 MAPK, PKG and JNKs with kinase inhibitors did not change sperm kinematics compared with the MLT group (Figures 6A–6C), implying that melatonin regulated sperm kinematics through the PKC signaling pathway. Then, we further inhibited melatonin receptors with luzindole and 4P-PDOT and detected PKC activity. The results showed that inhibition of the MT1 receptor significantly weakened PKC activity compared with that in the MLT group (Figure 6D), suggesting that PKC is downstream of MT1 and that melatonin regulates rabbit sperm kinematics via the MT1/PKC signaling pathway.

## DISCUSSION

To our knowledge, this study represents the first application of phosphoproteomics and upstream kinase prediction to investigate and elucidate the molecular mechanism by which melatonin influences rabbit sperm kinematics. Our findings provide evidence that melatonin regulates rabbit sperm kinematics through the MT1/PKC signaling pathway.

In the rabbit testes, AANAT, ASMT, MT1 and MT2 were mainly localized in the reproductive epithelium of seminiferous tubules, suggesting that melatonin might be synthesized in Sertoli cells and spermatogenic cells because AANAT and ASMT are the two key rate-limiting enzymes that synthesize melatonin. Studies have demonstrated the impact of melatonin on Sertoli cell metabolism [27], regulation of bovine Sertoli cell development and function [28], promotion of haploid germ cell development in Suffolk sheep [29], amelioration of restraint stress-induced oxidative stress and apoptosis in testicular cells [30], and protection of spermatogonia from oxidative stress [31], indicating that melatonin produced by the testes may affect and regulate spermatogenesis.

The positive impact of melatonin on sperm kinematics and semen quality has been observed in various species, including hamster [12], ram [13], stallion [15], boar [16], bull [32], and human [14], but prior to our study, not in rabbits. Results indicated enhanced rabbit sperm motility parameters with melatonin supplementation, manifested by increased plasma membrane integrity and mitochondrial membrane potentials, as well as reduced ROS. Melatonin accelerates rabbit sperm motility, potentially by partially eliminating reactive oxidative species and further enhancing mitochondrial activity.

The antiapoptotic effects of melatonin on ejaculated human spermatozoa are associated with the membrane melatonin receptor MT1 [20]. Research has also demonstrated that melatonin modulates ram sperm functionality via the activation of the MT2 receptor [22]. However, our results indicated that melatonin regulates rabbit sperm kinematics primarily through the MT1 receptor. MT1 and MT2 belong to the superfamily of guanine nucleotide-binding regulatory protein (G protein)-coupled receptors [33]. Melatonin combined with the MT1 or MT2 receptor and further activated G_i_ or G_q_ proteins. Once the G_i_ protein is activated, downstream kinase PKA may be inhibited. While the G_q_ protein is activated, downstream kinases PLC and PKC may be activated. Studies have shown that protein phosphorylation regulates sperm motility and kinematics [34,35]. Hence, phosphoproteomics of rabbit sperm was carried out to reveal the potential mechanism by which melatonin regulates sperm kinematics. The DE phosphoproteins for KEGG pathways were involved in sperm motility (ocu04714 thermogenesis) and glycolysis/gluconeogenesis (ocu00010), implying that melatonin may alter the phosphorylation of key proteins by the MT1 downstream signaling pathway, which may activate rabbit sperm kinematics as well as sperm metabolism. Glycolysis/gluconeogenesis further produces energy to support sperm motility [36].

Phosphopeptides obtained from rabbit sperm phosphoproteomics were analyzed by GPS 5.0 software to predict upstream protein kinases or phospholipases. We found that PKC, ERK1/2, p38 MAPK, PKG and JNKs are the top five enriched kinases, suggesting that these kinases downstream of MT1 may phosphorylate some pivotal proteins to regulate sperm flagellar movement. To uncover which signaling pathway worked in rabbit sperm, these kinase inhibitors were incubated with melatonin-treated rabbit sperm, and the results confirmed that the PKC signaling pathway plays an essential role in sperm kinematics. Moreover, we inhibited the melatonin receptors with luzindole or 4P-PDOT in melatonin-treated rabbit sperm and detected the PKC activity. We found that PKC activity markedly decreased compared with the melatonin group when the MT1 receptor was inhibited, implying that PKC is downstream of MT1 in response to melatonin in rabbit sperm.

**Figure 7:**
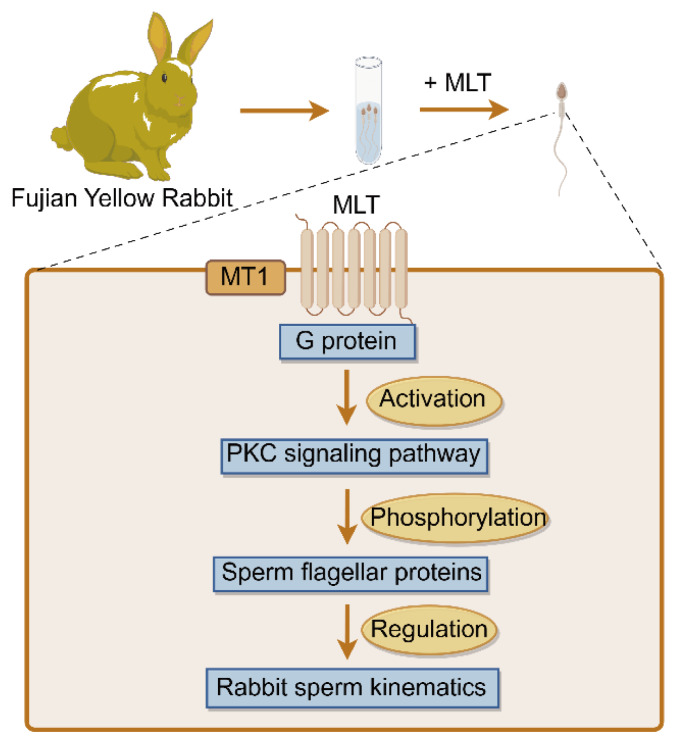
A brief proposal model of molecular mechanism that melatonin (MLT) regulates rabbit sperm motility and kinematics.

PKC is localized in the equatorial segment and the principal piece of the sperm tail [37], playing a crucial role as a signaling mediator associated with the regulation of flagellar motility [38]. Numerous studies have indicated that PKC kinase could phosphorylate proteins observed in sperm flagella and regulate their activity, which subsequently controls sperm motility [39,40]. Rabbit sperm phosphoproteomics also indicated that a large number of phosphorylated proteins are involved in sperm flagellar motility. Thus, the molecular mechanisms can be proposed to explain melatonin regulating rabbit sperm kinematics as follows: melatonin binds to the MT1 receptor and activates G protein, then further activates the PKC signaling pathway, which can probably phosphorylate sperm flagellar proteins and further regulate rabbit sperm kinematics (Figure 7).

In summary, the present study demonstrated the expression and localization of melatonin-related proteins in the rabbit epididymis and testes, the beneficial effects of melatonin on rabbit sperm, and the regulation of rabbit sperm kinematics by melatonin via the MT1/PKC signaling pathway.

## Figures and Tables

**Table 1:** Rabbit sperm motility and kinematics with different concentrations of melatonin for 60 min at 37°C

Parameters	0.1 mM	1 mM	5 mM	10 mM	BC	NC
TM (%)	77.53±1.00^b^	81.10±7.63^b^	90.27±0.47^a^	76.07±4.33^b^	83.33±1.12^b^	83.43±1.17^b^
PM (%)	70.80±2.32^ac^	73.50±8.57^a^	86.27±2.38^b^	62.97±8.70^a^	74.33±7.70^c^	74.20±3.44^c^
ALH (μm)	6.70±0.07^b^	6.63±0.19^b^	7.61±0.24^a^	7.02±0.33^b^	6.87±0.37^b^	6.68±0.28^b^
STR (%)	75.42±2.08^a^	77.40±0.20^a^	78.61±2.13^a^	76.03±2.73^a^	77.26±2.15^a^	76.72±1.55^a^
VAP (μm/s)	104.58±4.08^a^	105.24±3.44^a^	112.54±3.39^b^	95.50±3.88^c^	106.06±2.75^a^	104.96±3.30^a^
VCL (μm/s)	185.17±4.17^a^	183.09±1.72^a^	202.06±2.67^b^	185.16±3.13^a^	182.54±1.28^a^	183.79±3.75^a^
VSL (μm/s)	78.73±6.09^a^	81.31±3.47^a^	89.93±0.49^b^	73.85±6.41^a^	80.78±4.69^a^	80.33±6.30^a^
WOB (%)	56.59±2.27^a^	57.69±1.65^a^	56.34±2.77^a^	51.95±1.28^b^	57.58±1.14^a^	56.96±1.68^a^

a–c Different superscripts within the same row indicate statistical differences (p<0.05).

BC, blank control; NC, negative control; TM, the percentage of total motile sperm; PM, progressive motile sperm; ALH, amplitude of lateral head displacement; STR, straightness index; VAP, average path velocity; VCL, curvilinear velocity; VSL, straight-line velocity; WOB, wobble.

**Table 2:** Rabbit sperm motility and kinematics with different concentrations of melatonin for 90 min at 37°C

Parameters	0.1 mM	1 mM	5 mM	10 mM	BC	NC
TM (%)	79.63±3.13^a^	80.50±0.96^a^	86.07±0.61^b^	74.17±3.48^c^	80.17±1.28^ac^	77.63±1.89^c^
PM (%)	70.03±1.99^ab^	70.97±1.55^ab^	73.47±0.78^a^	58.73±4.75^c^	70.90±1.31^ab^	68.77±2.29^b^
ALH (μm)	6.73±0.24^a^	6.74±0.03^a^	7.79±0.35^c^	7.27±0.24^b^	7.05±0.19^a^	6.86±0.30^a^
STR (%)	72.23±0.64^a^	73.47±0.90^ab^	77.0±3.90^b^	76.36±3.94^ab^	72.55±1.62^ac^	71.71±1.67^ac^
VAP (μm/s)	97.95±8.62^ab^	97.41±5.99^ab^	98.96±3.88^a^	83.30±8.26^b^	93.05±8.27^ab^	92.49±12.71^ab^
VCL (μm/s)	179.16±2.97^a^	179.37±1.48^a^	191.58±6.58^b^	178.05±4.55^a^	179.76±1.10^a^	175.69±7.43^a^
VSL (μm/s)	72.00±6.24^ab^	72.74±5.83^ab^	78.86±4.47^a^	64.79±8.45^b^	68.93±7.92^ab^	67.28±10.46^ab^
WOB (%)	54.52±3.40^a^	54.53±2.64^a^	51.71±1.36^ab^	47.96±3.35^b^	51.90±3.99^ab^	52.63±5.02^ab^

a–c Different superscripts within the same row indicate statistical differences (p<0.05).

BC, blank control; NC, negative control; TM, the percentage of total motile sperm; PM, progressive motile sperm; ALH, amplitude of lateral head displacement; STR, straightness index; VAP, average path velocity; VCL, curvilinear velocity; VSL, straight-line velocity; WOB, wobble.
